# Evaluating the Adherence of Large Language Models to Surgical Guidelines: A Comparative Analysis of Chatbot Recommendations and North American Spine Society (NASS) Coverage Criteria

**DOI:** 10.7759/cureus.68521

**Published:** 2024-09-03

**Authors:** Advith Sarikonda, Emily Isch, Mitchell Self, Abhijeet Sambangi, Angeleah Carreras, Ahilan Sivaganesan, Jim Harrop, Jack Jallo

**Affiliations:** 1 Department of Neurological Surgery, Thomas Jefferson University, Philadelphia, USA; 2 Department of General Surgery, Division of Plastic Surgery, Thomas Jefferson University Hospital, Philadelphia, USA; 3 Department of Neurological Surgery, Thomas Jefferson University Hospital, Philadelphia, USA; 4 Department of Neurosurgery, Thomas Jefferson Medical College, Philadelphia, USA

**Keywords:** spine surgery, spine, guidelines, cervical fusion, large language models, chatgpt

## Abstract

Background

There has been a significant increase in cervical fusion procedures, both anterior and posterior, across the United States. Despite this upward trend, limited research exists on adherence to evidence-based medicine (EBM) guidelines for cervical fusion, highlighting a gap between recommended practices and surgeon preferences. Additionally, patients are increasingly utilizing large language models (LLMs) to aid in decision-making.

Methodology

This observational study evaluated the capacity of four LLMs, namely, Bard, BingAI, ChatGPT-3.5, and ChatGPT-4, to adhere to EBM guidelines, specifically the 2023 North American Spine Society (NASS) cervical fusion guidelines. Ten clinical vignettes were created based on NASS recommendations to determine when fusion was indicated. This novel approach assessed LLM performance in a clinical decision-making context without requiring institutional review board approval, as no human subjects were involved.

Results

No LLM achieved complete concordance with NASS guidelines, though ChatGPT-4 and Bing Chat exhibited the highest adherence at 60%. Discrepancies were notably observed in scenarios involving head-drop syndrome and pseudoarthrosis, where all LLMs failed to align with NASS recommendations. Additionally, only 25% of LLMs agreed with NASS guidelines for fusion in cases of cervical radiculopathy and as an adjunct to facet cyst resection.

Conclusions

The study underscores the need for improved LLM training on clinical guidelines and emphasizes the importance of considering the nuances of individual patient cases. While LLMs hold promise for enhancing guideline adherence in cervical fusion decision-making, their current performance indicates a need for further refinement and integration with clinical expertise to ensure optimal patient care. This study contributes to understanding the role of AI in healthcare, advocating for a balanced approach that leverages technological advancements while acknowledging the complexities of surgical decision-making.

## Introduction

Cervical fusion is a widely performed procedure with rapidly increasing usage. New York, for instance, saw a nearly twofold rise in annual cervical fusion rates from 1997 to 2012 [1]. Nationally, cervical fusion utilization rates, including anterior cervical discectomy and fusion and posterior cervical discectomy and fusion, are projected to substantially rise in the United States from 2020 to 2040 [2]. Despite this surge in utilization, there has been limited exploration into whether cervical fusion procedures align with evidence-based guidelines.

While numerous studies have investigated the implications of guideline adherence in lumbar fusions [3-6], the literature examining the same for cervical spine surgery remains scarce [3]. This paucity of research may be attributed to internal disparities among spine surgeons concerning their practice patterns and appropriate indications. Notably, studies have highlighted differences between orthopedic surgeons and neurosurgeons in various aspects of cervical fusion, encompassing indications, interpretations of imaging, and the utilization of external cervical orthoses post-surgery [7-9].

To address these issues, the North American Spine Society (NASS) has developed evidence-based medicine (EBM) coverage recommendations for various diagnostic procedures and therapeutic treatments in the field of spine surgery [10]. However, it has been noted that these guidelines are not consistently followed. Surgeon resistance to guideline adherence can be attributed to several factors, including a lack of awareness and familiarity with the guidelines, as well as perceived methodological inadequacies in research reports [11]. Furthermore, surgeons may believe that these guidelines do not accurately reflect the latest advancements in the field.

This paper explores the potential of large language models (LLMs) as tools for surgical decision-making, with a specific focus on cervical fusion procedures. Utilizing the updated 2023 NASS guidelines for cervical fusion as our reference, we formulated several clinical vignettes. These vignettes were then input into four prominent LLM tools to determine whether cervical fusion was warranted in each clinical scenario. Guideline adherence was assessed by evaluating the concordance of LLM responses with NASS recommendations. This study aims to shed light on the role of LLMs in healthcare, particularly their potential to standardize indications for cervical fusion.

## Materials and methods

Study design

This study employs a comprehensive observational approach aimed at evaluating the capacity of four LLMs, namely, Bard, BingAI, ChatGPT-3.5, and ChatGPT-4, to identify appropriate indications for cervical fusion based on clinical scenarios derived from the 2023 NASS guidelines. The primary objective is to assess each model’s adherence to these evidence-based guidelines, thereby exploring their potential utility in surgical decision-making.

The study design incorporates a series of 10 clinical vignettes, carefully crafted to represent a range of clinical situations where cervical fusion might be considered. These vignettes were explicitly developed based on the NASS guidelines to reflect common yet complex scenarios encountered in clinical practice. The inclusion criteria for the vignettes were guided by their relevance to the most up-to-date NASS coverage indications for cervical fusion, ensuring a robust assessment of the LLMs’ capabilities.

Ethical considerations

Given that this study exclusively involves the analysis of responses generated by artificial intelligence (AI) models and does not engage human subjects, it does not qualify as human subject research under federal regulations. Consequently, the study was exempt from the requirement for institutional review board approval. The ethical considerations primarily revolve around the responsible use of AI in clinical decision-making and ensuring that the findings are reported transparently and accurately.

Data collection and clinical vignettes

Data collection was performed in January 2024 using the following four LLM platforms: Bard (developed by Google), BingAI (developed by Microsoft), ChatGPT-3.5, and ChatGPT-4 (both developed by OpenAI). Each LLM was provided with 10 clinical vignettes, which included scenarios such as cervical spine tumors, traumatic injuries, deformities such as head-drop syndrome, and other conditions where the decision to perform cervical fusion is nuanced.

The vignettes were structured to capture a diverse set of clinical situations, each accompanied by a clear question regarding the indication for cervical fusion. For example, one vignette might describe a patient with cervical radiculopathy and query whether cervical fusion is recommended based on NASS guidelines. Each vignette was presented to the LLMs in a standardized format to minimize variability in the responses and ensure that the models’ outputs could be directly compared.

Response recording and processing

The responses generated by each LLM were meticulously recorded for further analysis. To ensure consistency and accuracy, each LLM was prompted multiple times if the initial response was unclear or lacked a definitive answer. For instance, if a model provided a conditional or non-committal answer, further prompts were used to elicit a clear yes or no response. If the model ultimately failed to provide a definitive answer, the response was categorized as “not definitive.” Each LLM response was generated with no more than two additional prompts, and all response times were immediate, ensuring efficient interaction during the evaluation process. This approach ensured that all responses were adequately captured and could be evaluated against the NASS guidelines.

Statistical analysis

The performance of LLMs was assessed using a combination of qualitative and quantitative methods. Descriptive statistics were employed to evaluate the overall concordance of each LLM’s recommendations with the 2023 NASS guidelines across all 10 clinical vignettes. Specifically, the proportion of correct responses, defined as those aligning with the NASS recommendations, was calculated for each LLM. This allowed for an initial comparison of the models’ overall accuracy.

For qualitative analysis, we conducted a thematic review of the errors made by the LLMs. This involved categorizing errors based on their nature (e.g., failure to recommend surgery when indicated, incorrect identification of contraindications) and identifying common patterns across the models. Additionally, scenarios where multiple models diverged from the NASS guidelines were analyzed in depth to elucidate potential limitations in the LLMs’ understanding of clinical nuances.

The results of both the quantitative and qualitative analyses are presented in detail in the tables in the Results section, with the quantitative findings highlighting the statistical significance of model performance differences and the qualitative analysis providing insights into common error patterns. These combined methods offer a comprehensive understanding of LLM performance in adhering to evidence-based clinical guidelines.

## Results

Of the 10 clinical vignettes, cervical fusion was indicated in seven (70%) cases according to NASS guideline recommendations (Table 1). Four scenarios received unanimous concordance with NASS guidelines. These scenarios were cervical spine tumor with anticipated instability following resection, cervical dislocation with a disability, cervical radiculopathy from isolated foraminal stenosis treated with partial medial foraminotomy, and cervical kyphosis with significant functional impairment. On the other extreme, all four LLMs were NASS-discordant for two vignettes, namely, head-drop syndrome with no functional limitations or documented progression of deformity, and pseudoarthrosis with no postoperative period of symptomatic relief. These results are summarized in Table 2. For all vignettes, ChatGPT-4 and Bing Chat achieved 60% NASS concordance compared to 50% concordance for ChatGPT-3.5 and Google Bard. Notably, no LLM achieved full concordance with NASS guidelines.

**Table 1:** NASS guideline recommendations for each clinical vignette. NASS: North American Spine Society

Clinical vignette	Does NASS recommend cervical fusion?	Official NASS Guideline [10]
A patient has an infection involving the spine in the form of osteomyelitis with the presence of instability. Is a cervical fusion indicated?	Yes	Cervical fusion is indicated for “infection (including tuberculosis) involving the spine in the form of discitis, osteomyelitis, or epidural abscess in EITHER of the following cases: a. Instability is present. b. Debridement and/or decompression is anticipated to result in instability” [10]
A patient has a tumor involving the cervical spine or spinal canal, resection and decompression of the spinal cord is anticipated to result in instability. Is a cervical fusion indicated?	Yes	Cervical fusion is indicated for “tumor involving the spine or spinal canal in EITHER of the following cases: a. Instability is present. b. Resection and/or decompression is anticipated to result in instability” [10]
A patient has a cervical dislocation with the presence of instability. Is a cervical fusion indicated?	Yes	Cervical fusion is indicated for “traumatic injuries, including fracture, fracture-dislocations, dislocations, or traumatic ligamentous disruption in EITHER of the following cases: a. Instability is present b. Decompression of the spinal canal is anticipated to result in instability” [10]
A patient has clinically significant head-drop syndrome which results in the inability of the patient to maintain a forward gaze. There is no documented progression of deformity, and the patient does not experience substantial functional limitations. Is a cervical fusion indicated for this patient?	Yes	Cervical fusion is indicated for “deformity that includes the cervical spine (e.g., kyphosis, head-drop syndrome, post-laminectomy deformity) that meets ANY of the following criteria: a. Clinically significant deformity that results in the inability of the patient to maintain a forward gaze. b. Substantial functional limitations including severe neck pain, difficulty ambulating, and decreased ability to perform activities of daily living c. Documented progression of deformity” [10]
A patient has cervical radiculopathy from isolated foraminal stenosis which was treated with a partial medial foraminotomy. Is a cervical fusion indicated for this patient?	No	Cervical fusion is not indicated for “cervical radiculopathy from isolated foraminal stenosis treated with a partial medial facetectomy/foraminotomy” [10]
A patient has cervical kyphosis and substantial functional limitations including severe neck pain, difficulty ambulating, and decreased ability to perform activities of daily living. Is a cervical fusion indicated?	Yes	Cervical fusion is indicated for “deformity that includes the cervical spine (e.g., kyphosis, head-drop syndrome, post-laminectomy deformity) that meets ANY of the following criteria: a. Clinically significant deformity that results in the inability of the patient to maintain a forward gaze. b. Substantial functional limitation including severe neck pain, difficulty ambulating, and decreased ability to perform activities of daily living. c. Documented progression of deformity” [10]
A patient has cervical myelopathy from a disc herniation, and I am planning a posterior laminectomy to decompress the spinal cord. Is a cervical fusion indicated as an adjunct treatment?	Yes	Cervical fusion is indicated for “cervical myelopathy (either from disc herniation, bony stenosis or ossification of the posterior longitudinal ligament (OPLL)) as an adjunct to decompression, that meet ANY of the following criteria: a. An anterior cervical discectomy or corpectomy is planned for decompression of the spinal cord. b. A posterior laminectomy is planned for decompression of the spinal cord” [10]
A patient has synovial facet cysts in the cervical spine, and I am planning to perform a cyst excision. Is a cervical fusion as an adjunct treatment indicated?	Yes	Cervical fusion is indicated for “synovial facet cysts in the cervical spine, as an adjunct to cyst excision.” [10]
A patient has pseudoarthrosis in the cervical spine. Imaging reveals intact instrumentation without screw breakage, screw loosening, or curve/correction decompensation. The patient has failed nonoperative care and has not had a period of time following index surgery during which they experience symptomatic relief. Is a cervical fusion indicated for this patient?	No	Cervical fusion is indicated for “pseudoarthrosis in the cervical spine that meets ALL of the following criteria (OR demonstrates the presence of a gross failure of the instrumentation (e.g., screw breakage, screw loosening, curve/correction decompensation): a. Postoperative onset of mechanical neck pain that is anatomically consistent with the level of the pseudarthrosis. b. A period of time following the index surgery during which the patient had symptomatic relief. c. Has failed nonoperative care. d. CT or plain films that are highly suggestive of nonunion at a motion segment at which a fusion had been previously attempted. These criteria include i. Lack of bridging bone. ii. Dynamic motion noted on flexion-extension-radiographs or from supine to standing radiographs” [10]. In this vignette, fusion was not indicated because the patient did not have a period of time following index surgery during which they experienced symptomatic relief
A patient has cervical radiculopathy due to bony stenosis. The patient has trialed nonoperative treatment for five weeks. Due to the severity of symptoms, the patient is unable to work and is experiencing functionally limiting weakness. Is a cervical fusion indicated for this patient?	No	Cervical fusion is indicated for “cervical radiculopathy from degenerative disorders (either from disc herniation or bony stenosis), as an adjunct to disc excision, that meet ALL of the following criteria: a. Pattern of radiculopathy explained by imaging. b. Six to 12 weeks of an appropriate course of nonoperative treatment. c. The following can mitigate the need for an initial nonoperative trial: i. The severity of symptoms prevents the patient from working. ii. Functionally limiting motor weakness” [10]. In this vignette, fusion was not indicated because the patients trialed nonoperative treatment for five weeks, instead of six to 12 weeks

**Table 2:** NASS concordance for each large language model. NASS: North American Spine Society

Topic of clinical vignette	Is cervical fusion indicated?					Concordance with NASS guidelines (n, %)
	NASS	ChatGPT 3.5	ChatGPT 4	Google Bard	Bing Chat	
Osteomyelitis with instability	Yes	Yes	Yes	No	Yes	3/4 (75%)
Cervical spine tumor with anticipated instability following resection	Yes	Yes	Yes	Yes	Yes	4/4 (100%)
Cervical dislocation with instability	Yes	Yes	Yes	Yes	Yes	4/4 (100%)
Cervical deformity (head-drop syndrome)	Yes	No	No	No	No.	0/4 (0%)
Cervical radiculopathy from isolated foraminal stenosis treated with partial medial foraminotomy	No	No	No	No	No	4/4 (100%)
Cervical kyphosis with significant functional impairment	Yes	Yes	Yes	Yes	Yes	4/4 (100%)
Fusion as an adjunct to posterior laminectomy to decompress cervical spine	Yes	No	Yes	No.	Not definitive	2/4 (50%)
Fusion as an adjunct to synovial facet cyst resection	Yes	No	No	No	Yes	1/4 (25%)
Pseudoarthrosis in the cervical spine with no postoperative period of symptomatic relief	No	Yes	Yes	Yes	Yes	0/4 (0%)
Cervical radiculopathy due to bony stenosis with nonoperative treatment for five weeks	No	Yes	Yes	No	Yes	1/4 (25%)
Concordance with NASS guideline recommendation (n, %)		5/10 (50%)	6/10 (60%)	5/10 (50%)	6/10 (60%)	

## Discussion

The NASS guidelines originally emerged as part of an initiative to introduce Appropriate Use Criteria (AUCs) into spine surgery [10,12]. AUCs are developed through clinical scenarios presented to a multidisciplinary panel of physicians. While it is crucial to emphasize that the NASS guidelines are not designed to replace physician decision-making, they do offer a valuable framework grounded in evidence-based medicine.

LLMs have been proposed for use in various surgical contexts, encompassing surgical planning, diagnosis, and patient care [13-16]. Notably, substantial differences in concordance with clinical guidelines have been observed among different LLMs. For instance, a study by Duey et al. demonstrated that ChatGPT-3.5 exhibited concordance with NASS guidelines for thromboembolic prophylaxis in only 33% of prompted vignettes, in contrast to ChatGPT-4, which achieved 92% concordance [17]. Recognizing this variability, we posed our clinical vignettes to multiple popular LLM chatbots. Regrettably, none of the chatbots included in our analysis achieved a concordance rate exceeding 50% with NASS guidelines.

Interpretation of results

The guidelines provided by the NASS recommend fusion as an adjunct to posterior laminectomy for decompressing the cervical spine. However, these guidelines do not specify the number of decompressed levels, which might be considered a limitation. Surgeons may question whether a single-level decompression procedure warrants a follow-up fusion, potentially explaining why only one LLM out of the four (ChatGPT-4) concurred with NASS guidelines in this particular scenario.

Google Bard, in response to the vignette, provided two reasons against fusion. First, they suggested that fusion might introduce unnecessary risks and potentially limit mobility. Second, they noted that fusion is not always necessary for stability, citing studies indicating good long-term stability outcomes with isolated laminectomy for cervical myelopathy from disc herniation, particularly in single-level procedures.

Similarly, fusion as an adjunct to facet cyst resection also received only one NASS-concordant response, which came from Bing Chat. NASS discourages the removal of cervical facet cysts through decompression alone, citing a high incidence of post-laminectomy kyphosis. However, prior research has reported comparable outcomes for patients who undergo decompression alone versus decompression and fusion in treating cervical facet cysts [18]. Unlike lumbar synovial cyst treatment [19,20], there is limited literature addressing cervical cyst treatment, potentially explaining the lack of fusion recommendations from most LLMs.

As previously mentioned, our clinical vignette involving a patient with head-drop syndrome did not receive any NASS-concordant responses from the LLMs. In this scenario, the patient’s head-drop syndrome affected their ability to maintain a forward gaze. However, there was no documented progression of deformity, and the patient did not experience substantial functional limitations, such as severe neck pain, ambulation difficulties, or impaired activities of daily living. ChatGPT-4 argued that fusion in such cases might constitute potential overtreatment due to the absence of progressive deformity. However, according to NASS guidelines, fusion is indicated if the patient experiences a “clinically significant” deformity that limits forward gaze, even in the absence of severe functional impairments or documented progression of the deformity.

Another vignette that received 0% NASS concordance from LLMs involved a patient with pseudoarthrosis and intact instrumentation on imaging. This patient had failed nonoperative care and had not experienced symptomatic relief following the index surgery. According to NASS guidelines, cervical fusion is indicated for such patients if they meet specific criteria, including postoperative onset of mechanical neck pain consistent with the level of the pseudoarthrosis, a period of time after the index surgery during which the patient had symptomatic relief, failure of nonoperative care, and imaging suggestive of nonunion at a previously fused site. In this case, fusion was not indicated because the patient did not experience symptomatic relief following the index surgery.

It is essential to acknowledge that LLMs, such as ChatGPT and Bard, rely on preexisting internet text, making responses to nuanced questions, especially those with limited supporting literature, potentially inconsistent or inaccurate. As previously highlighted, the indications for cervical fusion exhibit substantial variability, both among neurosurgeons and spine surgeons in general.

In the context of AI and LLMs, utilizing highly nuanced and challenging prompts is crucial for several reasons. First, it enhances the robustness and adaptability of the models. LLMs are typically trained on vast datasets comprising diverse text from the internet, and while they can generate coherent responses, their proficiency often diminishes when faced with complex, context-specific queries. Difficult prompts push these models to navigate intricate linguistic, contextual, and semantic nuances, thereby testing and expanding their boundaries. This process is akin to advanced problem-solving exercises in human education, where tackling challenging problems helps deepen understanding and improve problem-solving skills.

Moreover, the use of nuanced prompts is beneficial for refining the models’ decision-making capabilities in specialized fields, such as healthcare. These vignettes encapsulated real-world complexity by incorporating multifaceted clinical scenarios, thereby providing a rigorous testing ground for the LLMs. This not only highlighted the current limitations in the models’ ability to adhere to clinical guidelines but also pinpointed specific areas needing improvement, such as the interpretation of head-drop syndrome and pseudoarthrosis cases.

Furthermore, difficult prompts can reveal the inherent biases and gaps in the training data of LLMs. By presenting scenarios that require an understanding of less common or more controversial aspects of a domain, researchers can identify where the models may fall short or exhibit biases. For instance, discrepancies observed in LLM responses to cervical fusion indications might reflect underlying gaps in the training data related to spine surgery guidelines. Addressing these gaps through targeted training and prompt refinement can lead to more accurate and reliable model outputs. The deliberate use of challenging and nuanced prompts in testing LLMs is a strategic approach to enhance their robustness, adaptability, and domain-specific expertise. It not only helps in identifying and addressing the models’ limitations but also contributes to the continuous improvement of AI systems, making them more reliable and effective tools in specialized fields such as healthcare.

While EBM guidelines such as those from the NASS provide a structured framework for clinical decision-making, not all surgeons uniformly adhere to these recommendations. There is a recognized divergence in practice patterns among surgeons, often influenced by their clinical experience, personal judgment, and interpretations of individual patient cases. For instance, in the realm of cervical spine surgery, some surgeons might opt to perform fusion procedures even when not strictly indicated by NASS guidelines. These decisions can stem from a variety of factors, including perceived benefits to the patient’s quality of life, the surgeon’s assessment of potential future complications, or a more conservative approach to patient care. Differences in training backgrounds, subspecialty focuses, and evolving clinical evidence also contribute to these variations. Consequently, while guidelines aim to standardize care and improve outcomes, the nuanced nature of clinical practice means that deviations occur as surgeons weigh the guidelines against their clinical expertise and patient-specific factors. This underscores the complexity of surgical decision-making and the importance of integrating EBM guidelines with personalized patient care.

Future directions

Future directions for studies evaluating the adherence of LLMs to clinical guidelines, such as those for cervical fusion, should focus on several key areas to enhance their relevance and accuracy. First, expanding the scope of clinical scenarios to include a broader range of patient conditions and comorbidities will provide a more comprehensive assessment of LLM performance. Incorporating multimodal inputs, such as medical imaging and laboratory results, could also significantly improve the realism and applicability of the study, as these elements are integral to clinical decision-making. Additionally, developing methodologies to assess the ability of LLMs to provide rationale for their recommendations can offer insights into their decision-making processes and identify areas needing refinement. Another promising direction is the integration of continuous learning frameworks, where LLMs are regularly updated with the latest clinical guidelines and medical literature, ensuring their recommendations remain current and evidence-based. Collaborative efforts between AI developers, clinicians, and researchers can further facilitate the creation of more sophisticated models tailored to the complexities of healthcare. Finally, investigating the impact of LLM-assisted decision-making on clinical outcomes through prospective studies will be crucial in validating their practical utility and safety in real-world settings.

Limitations

Our study presents several limitations that merit consideration. First, the NASS document outlining cervical fusion indications encompasses only 11 guideline recommendations. This limited set may not fully capture the diverse spectrum of patients presenting with cervical spine pathologies, particularly those with multiple comorbidities. Notably, cervical spine degeneration has been associated with conditions such as cancer, depression, and hypertension, none of which are addressed in the NASS guidelines [21]. Additionally, the guidelines do not account for social determinants of health, which can significantly influence the treatment patients ultimately receive.

Furthermore, it is critical to acknowledge that LLMs such as ChatGPT and Google Bard are not inherently designed to provide binary yes/no responses to specific clinical questions such as “Is cervical fusion indicated for this patient?” As a result, we had to structure our prompts in a manner that requested only a “yes or no” answer to fit our clinical vignettes.

Another significant limitation is the absence of imaging in our study’s prompts. In clinical practice, medical imaging plays a crucial role in decision-making. The omission of imaging data does not fully replicate the complexities of real-world clinical scenarios and may affect the accuracy of LLM responses.

Moreover, the variability in LLM performance highlights the need for more nuanced training data that includes a wider array of clinical scenarios and guideline interpretations. This would better equip the models to handle the intricacies of surgical decision-making.

## Conclusions

Our research significantly contributes to the ongoing dialogue on integrating LLMs into healthcare decision-making. While these models show great promise in standardizing and refining indications for cervical fusion procedures, it is essential to recognize the associated challenges and limitations. The inherent subjectivity of surgical decision-making, the necessity for comprehensive and detailed guidelines, and the importance of highly contextualized prompts are crucial factors that should shape future research. Addressing these elements will enhance the models’ ability to support patient care effectively. Ultimately, the synergy between clinical expertise and advanced AI tools holds the potential to foster more precise, evidence-based, and patient-centric healthcare practices.
